# Statistical inference links data and theory in network science

**DOI:** 10.1038/s41467-022-34267-9

**Published:** 2022-11-10

**Authors:** Leto Peel, Tiago P. Peixoto, Manlio De Domenico

**Affiliations:** 1 Department of Data Analytics and Digitalisation, Maastricht University, Tongersestraat 53, 6211 LM Maastricht, The Netherlands; 2 Department of Network and Data Science, Central European University, 1100 Vienna, Austria; 3 Department of Physics and Astronomy ‘Galileo Galilei’, University of Padua, Via F. Marzolo 8, 35126 Padova, Italy

**Keywords:** Applied mathematics, Complex networks

## Abstract

The number of network science applications across many different fields has been rapidly increasing. Surprisingly, the development of theory and domain-specific applications often occur in isolation, risking an effective disconnect between theoretical and methodological advances and the way network science is employed in practice. Here we address this risk constructively, discussing good practices to guarantee more successful applications and reproducible results. We endorse designing statistically grounded methodologies to address challenges in network science. This approach allows one to explain observational data in terms of generative models, naturally deal with intrinsic uncertainties, and strengthen the link between theory and applications.

## Abstract (web summary)

Theoretical models and structures recovered from measured data serve for analysis of complex networks. The authors discuss here existing gaps between theoretical methods and real-world applied networks, and potential ways to improve the interplay between theory and applications.

## Introduction

Network science is the study of complex systems composed of fundamental units, represented as nodes, and their interactions or relations, represented as links^1,2^. This mathematical abstraction presents the opportunity to study an interacting system as a whole, and allows us to uncover important insights that we would otherwise miss had we studied the system as a simple collection of units, e.g., by reducing it to the sum of its parts or any other naive aggregation. Furthermore, network science points towards a unified formalism that can be used to describe systems belonging to various scientific domains, but can nevertheless be cast as a network of interacting elements. Indeed, this holistic treatment had been employed for the study of a wide variety of complex systems, including biological (from cellular biology^3–5^ to neural circuits^6–8^ and ecological food webs^9–11^), technological (from coupled infrastructures^12,13^ to communication systems^14,15^) and social (from socio-technical relationships^16–19^ to animal interactions^20,21^) systems, enhancing our understanding of emergent phenomena in society^22,23^, life^24–26^ and disease^27–29^, as well as their combination^30–35^.

Despite the comprehensive embrace of this holistic ethos in such a large variety of scientific disciplines, it is perhaps surprising to find that the development of network science theory and its application to specific domains often occur with some degree of isolation. Consequences of this separation are (i) that general network science methods are typically developed that do not consider the provenance of the underlying data and (ii) that network science methods are misused and/or used out of context. These issues arise because, on the one hand, methods are often developed and tested with little consideration for inherent uncertainties and incompleteness of readily-available empirical data, or even an in-depth understanding of its provenance and measurement procedure. On the other hand, original empirical work that involves collecting data and subsequent analysis, often employs off-the-shelf methodology that may be incompatible with the data and/or domain. Perhaps this separation of tasks can be considered a simple and convenient division of labour that naturally occurs as individual researchers specialise within a given domain. One might argue that such specialisation is reasonable, offering a more efficient path to solving specific challenges and even that it is necessary to flourish within the competitive environment of scientific research.

Here we highlight some of the risks associated with such a separation within a field as diverse and interdisciplinary as network science. The variety of potential problems, range from the proliferation of theories that lose sight of target research questions to applying methods while overlooking the underlying hypotheses and limitations. The use of inappropriate methodology or blind application of methods in lieu of a clear understanding runs the risk of cutting down network science before it has the chance to fully bloom, and can undermine the efforts for dialog and confrontation built over more than two decades by those scientists who are working from theoretical to applied problems.

## Linking data and theory in network science

The central issue we wish to highlight is that, when manifest, the disconnect between method and application erases the crucial distinction between data (i.e., what is actually measured) and abstraction (i.e., the underlying network representation). Very often, researchers choose to draw the shortest line from the measurements at hand to a network representation, ignoring the fact that the actual interactions between network elements usually manifest themselves only indirectly in the observations. This occurs for a variety of reasons, ranging from uncertainties in the measurements, data inaccessibility or inappropriate choice of representation.

Here we argue that the most appropriate stance to take is to frame network analysis as a problem of *inference*, where the actual network abstraction is hidden from view, and needs to be reconstructed given indirect data. We illustrate this in Box 1, where we show some examples of indirect measurements—incomplete and erroneous networks, time series dynamics and proximity events—that point only indirectly at the underlying network structure, which needs to be reconstructed.

In this work, we will focus on three intimately related factors that are often overlooked as a consequence of the aforementioned gap between theory and practice in network science: (1) The obscured quality of the data. (2) The choice of representation. (3) The suitability of the methods. We argue that in order to close this gap it is necessary to adopt a model-centric approach to data-driven network analysis, requiring an appropriate level of abstraction to describe the object of study (often not directly observed in the data), and an inferential step that allows us to extract that abstraction from the (potentially indirect) data at hand. The methodology providing this connection should be derived from first principles, and should be tailored to particular domains of application.

We will demonstrate typical pitfalls that result from blurring the distinction between data and abstraction, recent work that has been done to address them, as well as existing challenges and unexplored open problems. We focus primarily on the structural analysis of networks, as determining the network structure is typically the first step in any network analysis. However, the points we raise apply equally to the analysis of empirical measurements *on* networks, i.e., dynamical processes that take place on them.

Box 1 Linking data and theory in network science

A network of interactions ***A*** that gives as a result some kind of observational data ***D*** should not in general be conflated with the data itself. Instead, we need to recognize that the data ***D*** is the result of measurement process *P*(***D***∣***A***) that is conditioned on the unseen network, but is to some extent unavoidably decoupled from it. In order to estimate the underlying network, we need to perform an inferential step *P*(***A***∣***D***), which needs to include our modeling assumptions about how the network and the data are generated. The resulting estimate ${{{\hat{{{{{\boldsymbol{A}}}}}}}}}$ will have an uncertainty that reflects the experimental design, accuracy of the measurements and overall feasibility of the particular reconstruction problem.

## Obscured quality of data

When network data are considered, measurement uncertainties are almost universally neglected, and empirical studies are frequently carried out as if the given network representation of a real system is perfectly accurate — an untenable practice long discarded in established empirical fields. Observational data is typically incomplete, for practical reasons, which should raise questions about whether the dataset is representative or biased and to what extent. Just to give brief examples, respondents in survey data may interpret questions in different ways, thus potentially generating inconsistencies^36^, and observations of interactions between plant and pollinator species may miss pollination contacts either due to chance^37^ or because some species are more difficult to spot than others. Furthermore, even if the process of data collection is accurate enough such that these uncertainties could be neglected, there are always possibilities for the inclusion of transcription or copying errors. It might seem trivial to an experimentalist, but the heterogeneity of backgrounds in Network Science means that these issues are recognized potential as problems to varying extents and often overlooked entirely.

A case in point is the Zachary’s Karate Club network, which represents the social interactions between members of a karate club^38^. This network is one of the simplest and most widely used social networks for developing Network Science methods—most often employed only as a standard test network, rather than an object of study in its own right. It is worth mentioning an obvious transcription error in the paper that prevents a fully unambiguous accounting of the data: the recorded adjacency matrix contains a provable error as the entry (23, 34) is zero while the entry (34, 23) is nonzero even though the network is undirected. This ambiguity occurs in both the weighted and unweighted versions of the matrix presented in the original paper. Furthermore, even if interactions among people had been manually recorded by Zachary in completeness without errors, the underlying raw data (direct observations, surveys, school records) and their associated uncertainty are unavailable to the research community. This lack of fundamental measurement information severely limits what can be ultimately learned from this data.

The possibility of measurement errors and omissions should be considered the rule, rather than the exception, whenever data about the real world is collected. Typically, those researchers who work within a specific application area know well these issues and how they manifest within their specific domain. For instance, the computational social scientist studying human behavior on social media will be aware that the data they receive from the platform API is a potentially biased subset of a complete dataset^39^ and that the set of users of a specific platform are not necessarily representative of the general population^40^. However, such specific domain knowledge is rarely captured in the data that are pre-processed and made publicly available—the data upon which the computational tools of network science are developed. The absence of this metadata that describes the quality of the original data means that many of the tools of Network Science are built upon the implicit hypothesis that networks have been constructed perfectly without errors or omissions in the data—something that rarely can be justified. Because of this, the rather uncontroversial fact that all data contain some amount of uncertainty is almost never incorporated into network analyses and their conclusions, and any awareness of this fact is (at best) left only as a *post hoc* admonition or disclaimer.

**Fig. 1:**
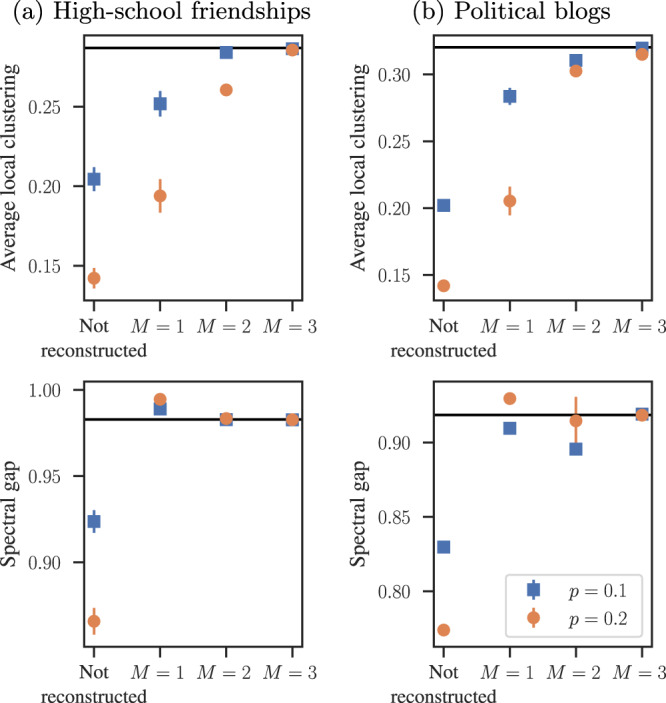
Network descriptors measured from noisy networks and their reconstruction. We investigate two empirical networks: **a** friendships between high school students and **b** hyperlinks between political blogs—measured with a simulated missing edge probability *p*, and spurious edge probability $$q=p{E}\bigg/\left[\left(\begin{array}{c}N\\ 2\end{array}\right)-E\right]$$. We consider the resulting network as it is measured in this noisy manner (“not reconstructed”), and the reconstructed networks obtained with the Bayesian method of ref. 53, after *M* independent noisy measurements are performed. We show the values of averaged local clustering coefficient and spectral gap of the corresponding random walk transition matrix, obtained in each case. The error bars correspond to the standard deviation of posterior distribution. The horizontal line shows the true values for the original network.

The central issue here is not accuracy itself, but the lack of error assessment. Network data are not inherently less or more accurate than any other kind of empirical data. However, the errors in network data can easily be amplified in network analyses, and in manners that are difficult to predict due to the highly nonlinear nature of how they depend on the network structure, making it hard to estimate the uncertainty of our final conclusions. Figure 1 illustrates this problem, in which we compute commonly used network descriptors for a simulated noisy measurement of two empirical networks, one of friendships between high-school students^41^, and the other of hyperlink connections between political blogs^42^. These networks themselves are measured and therefore contain their own unknown errors. However, for the purpose of this simulation, we assume they are complete without any errors. Based on this fictitious “true” network, we simulate an edge not being recorded (a “false negative”) with a uniform probability *p* and a non-edge being recorded as an edge (a “false positive”) with a probability $$q=pE/[\left(\begin{array}{c}N\\ 2\end{array}\right)-E]$$, where *N* and *E* are the number of nodes and edges in the original network. The probability *q* is chosen in this way such that the expected number of observed edges is the same as in the original network. One might naively expect that the effect of this kind of uniform measurement error is simply to introduce fluctuations around the values of the network descriptors for the original network. Instead we observe systematic biases that shift these values into particular directions such that they no longer resemble those of the original network and, importantly, they can hide existing structures or amplify existing ones.

It should be remarked here that early work in network science acknowledged that network data are susceptible to errors^43^ and laid the foundations for the task of inferring missing links from an observed network that would later be known as link prediction^44^. While Goldberg and Roth^43^ developed heuristics to leverage the newly discovered “small-world” properties of networks^45^, others used node attributes to predict missing links^46,47^. Since then a numerous literature on the topic of link prediction^44,48–50^ has emerged, including the related, but distinct, task of predicting future links in temporally evolving networks^51^. However, it appears as though these works have been developed within their own silo of “link prediction” with little interaction with the rest of network analysis (notable exceptions are the works of Clauset et al.^49^ and Guimerá et al.^48^, who framed link prediction as a statistical inference problem, based on generative network models). Decomposing a problem into separate subtasks can offer great benefits in how we organize scientific development, but this separation incurs the risk of these subtasks becoming universes in their own right, and the fact that they are a piece a larger puzzle can easily be forgotten. In the specific case of link prediction, researchers tend to focus on the core theoretical problem but often neglect essential practical matters, e.g., most emphasis is given to ranking edges according to those most likely to be present, but not to how many edges should be predicted or the impact of asymmetries in misclassification costs. Framing link prediction as a ranking problem allows different methods to be easily compared to each other, but it does not by itself deliver a complete methodology that can be readily used by practitioners. Because of this, the abundant literature on link prediction has not resulted in an abundance of actual links predicted.

Fortunately, more recently the idea of uncertain network data has been restored and started to return to the mainstream with a few researchers bringing forth a research agenda devoted to the incorporation, both theoretically and in practice, of the existence and the effects of errors in network measurements. The first step in this direction is to embrace the fundamental distinction between data and abstraction, and accept the fact that whatever measurement we make is not necessarily "the” network we want to study, but includes at least some amount of distortion. Consequently, studying a network should be framed in terms of *network reconstruction*, which we have already introduced in Box 1. This idea is implemented as quantitative methodology by formulating generative models that combine both the noisy measurement, expressed as a probability *P*(***D***∣***A***) of observing data ***D*** given an underlying network ***A***, as well as the latent network structure, encoded in a prior probability *P*(***A***), and using this information to determine which are the networks that are more likely to lie behind a given observation, via the Bayesian posterior distribution

1 $$P({{{{{{{\boldsymbol{A}}}}}}}}|{{{{{{{\boldsymbol{D}}}}}}}})=\frac{P({{{{{{{\boldsymbol{D}}}}}}}}|{{{{{{{\boldsymbol{A}}}}}}}})P({{{{{{{\boldsymbol{A}}}}}}}})}{P({{{{{{{\boldsymbol{D}}}}}}}})}.$$

This approach is arbitrarily extensible, since we can replace our measurement models and network prior as desired, incorporating appropriate domain knowledge, to better suit any particular application.

In the context of social networks, Butts^36^ has implemented the above approach to take into account reporting errors in network data. Newman^52^ has also used the above approach to show how multiple independent network measurements can be used to reconstruct a network that is more accurate than what we would obtain with individual measurement. Building upon the seminal works of Guimera et al.^48^, Clauset et al.^49^ and Airoldi et al.^50^, Peixoto^53^ extended this framework by introducing structured network priors that can uncover the latent modular network structure, and use this information to determine what is more likely to be the underlying network, even in situations where a single network measurement is made, and the errors are unknown. Young et al.^54^ built a general setup where the practitioner can specify an arbitrary measurement model, in a situation where multiple measurements are made.

Figure 1 also shows the results obtained with the reconstructed networks using the method of ref. 53, which are closer to the true values (even for single measurements, *M* = 1), and approaches them asymptotically as the number of independent measurements increases. These kinds of result illustrate two main points: i) More sensible approaches that incorporate, rather than ignore, the possibility of measurement error can improve the analysis even when information about the uncertainty of the data is unavailable; ii) Accurate results are only ultimately possible if proper error quantification is made (as in the multiple measurements *M* > 1 in Fig. 1), and these are taken into account in the analysis. Although there are already methods developed that implement the first approach, these can still be further developed, incorporating more realistic and diverse network models, and be made more computationally efficient. However, these approaches will invariably hit upon fundamental limits of reconstruction, which can only be lifted if proper error quantification is incorporated in the data acquisition phase. This means that accurate network analysis will only be possible if the empirical practice in network science ceases to omit such crucial information.

Although still far from common practice, we see that the network science community has at least considered issues of data uncertainty and the growing emphasis on network reconstruction is promising. However, it is important to note that not only links but also the nodes (and their attributes, or metadata) may also contain errors, which are often seen in the context of node identity. For instance, consider a collaboration network in which nodes are authors who are linked if they have worked together. These networks can be constructed from bibliometric data, but defining a node requires matching an individual author across multiple publication records. Errors can easily arise because different authors can have the same name, potentially causing multiple nodes to be collapsed into a single node, and an individual author can have different collaborators and institutional affiliations, or a name that appears under multiple spellings, potentially representing a single node as multiple nodes. These issues are further exacerbated by errors or differences in recording node attributes, e.g., author affiliations are often formatted differently from journal to journal.

This kind of node uncertainty has not really received sufficient attention, yet, in the network science literature. However, resolving these uncertainties has been previously explored in the context of matching entities across different data sources^55,56^. The problem is commonly referred to entity resolution but also known by other names such as record linkage, deduplication, object identification and identity uncertainty, among others. Although much of the work in this area was not applied to data that we typically represent as a network, later works leveraged the network structure in order to improve matches^57–59^.

## Choice of representation

The choice of relevant variables is one of the most difficult tasks in any scientific discipline, and it might be even more problematic in network science, since it deals with complex systems that admit multiple representations.

When conducting network analysis, it should be common practice to carefully think about what are the nodes and what are the edges. This issue has been made explicit by Butts^60^, who warned about how defining what is a node and an edge is a central choice with crucial consequences to the network analysis. Butts argued that since the network representation is always an approximation of the underlying system, its construction is a theoretical act, that cannot be free of assumptions, which in turn need to be made explicit and scrutinized.

As we have already mentioned, it is not difficult to confuse a particular mathematical description of a network with the actual underlying object of inquiry. In fact, it is common practice to draw the shortest route from the data at hand to a network representation, often referred to as “the” network, even if in many cases this might be ignoring the crucial distinction between data and abstraction, for which there are many alternatives that should be at least inspected before jumping into the analytical stage. In fact, the omission of measurement errors discussed above can be seen as a particular symptom of this shortsightedness, but the underlying problem too often goes well beyond this. As a simple example, we can take *any* network and transform it into its corresponding line graph by replacing the edges with nodes, significantly changing the properties of the network^61,62^. From a purely mathematical point of view, neither representation can be considered “the” network, since this transformation is reversible. Although in most contexts the situation is far less ambiguous than this, nevertheless it is less common to consider the possibility that an observed pairwise relation in some data should be better understood as an indirect manifestation of some hidden relationship, which may involve different units altogether, or even none at all.

Once again, we can use the Zachary’s Karate Club as a simple example. In the original paper the reported network is based upon some unknown function of multiple types of observed social interaction, that hide, in fact, the underlying chosen representation. What does each link in Zachary’s network really tell us about the social interactions among the members of the Karate club? Not knowing precisely what has been measured by Zachary, and how, prevents any real scientific understanding to be extracted from what has been reported.

We can solve a large part of this issue with the choice of representation once we require our network abstraction to be part of a *generative process* — a mathematical model that is capable of reproducing, at least in principle, whatever data ***D*** we are observing, conditioned on the unseen network ***A*** as an unknown parameter. This takes the form of the probability *P*(***D***∣***A***) in Box 1. Arguably, it is only when such a model is actually elaborated that we finally have a precise definition of what our network abstraction means and how it relates to the data. After the generative model is decided, the inference procedure that reconstructs the network follows directly from the posterior distribution *P*(***A***∣***D***), given by Eq. (1), in a principled manner. What remains are theoretical and technical issues relating to the tractability, efficiency and feasibility of the inference, but no longer its interpretability. The uncertainty of the estimates and statistical significance of the ensuing analysis follow inherently as a natural consequence of this approach.

In the following, we illustrate common pitfalls that arise from ignoring or avoiding the above prescription.

(I) Reconstruction based on correlations and thresholding. We argue that it is crucial to understand not only the provenance of network data, but also to make explicit the underlying abstraction that we wish to extract from it. Perhaps the best systematic example of this problem is the study of time-varying signals, where each node produces a time series, the underlying network structure — responsible for coupling the dynamics of distinct nodes — is unknown and the shortest line from data to network is to consider the correlation between time series as the “edges” of a weighted “correlation network”. However, it is elementary that correlation is fundamentally different from causation, and the observed correlation in many cases is a result of indirect causal relationships between the units, or in fact by no causal relationship at all.

One may argue that although not sufficient, correlation is necessary evidence of causation, and therefore identifying patterns of correlation may yield insights about the underlying causal relationships. Indeed, with purely observational data (i.e., with no ability to perform interventions) there may be ultimately no alternative^63^. However, approaching this problem naively is a reason of high concern. We illustrate this with a deeply flawed, but commonly used approach of extracting associations based on correlations, where an association between two time series is deemed to exist if their correlation exceeds a predefined threshold. We can see how this fails with a basic example: Consider three variables *X*(*t*), *Y*(*t*) and *Z*(*t*) that each represent time series in which *X*(*t*) and *Y*(*t*) are simply noisy perturbations of *Z*(*t*), e.g.,

2 $$\begin{array}{rcl}X(t)&=&Z(t)+{\epsilon }_{1}\\ Y(t)&=&Z(t)+{\epsilon }_{2},\end{array}$$

where *ϵ*_1_ and *ϵ*_2_ are independent noise terms. Clearly, *X*(*t*) and *Y*(*t*) are conditionally independent given *Z*(*t*), but *X*(*t*) and *Y*(*t*) will have a nonzero correlation that can exceed any arbitrary threshold. Therefore, correlation thresholding will impute that an edge exists between *X*(*t*) and *Y*(*t*), even though none exists.

**Fig. 2:**
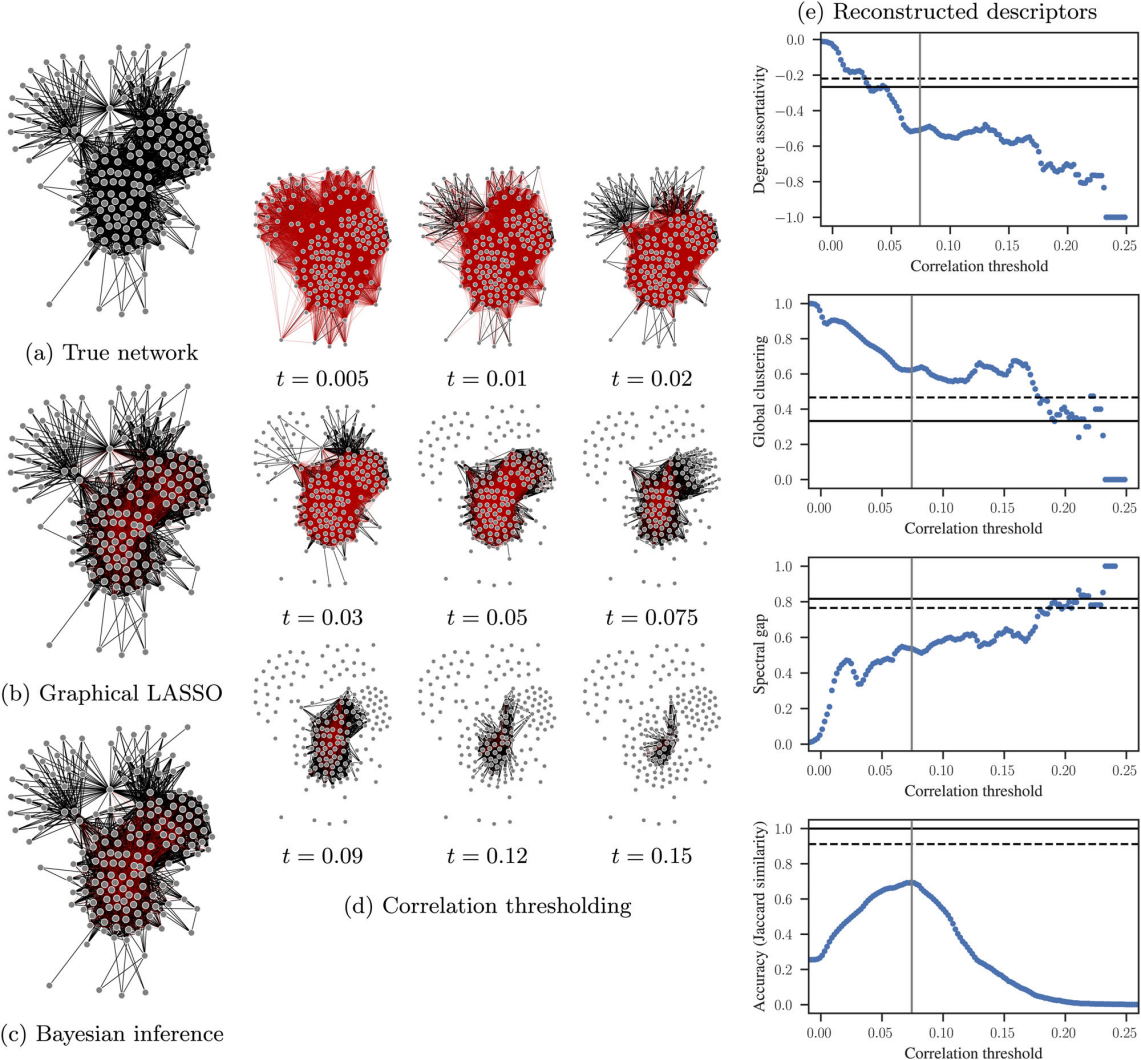
Network reconstruction in action. Reconstruction of an empirical food web (upper left), from *M* = 10^5^ samples of the Ising model at critical temperature. *Left panels:* **a** The true original network, compared with a reconstructed network obtained by inferring a sparse precision (inverse covariance) matrix using the graphical LASSO (**b**). Edges that do not exist in the true network are colored red. The graphical LASSO assumes the data is drawn from a multivariate Gaussian, which is clearly a misspecification. However, even this misspecified model performs better than naive correlation thresholding. The result obtained with the Bayesian method of ref. 68, which matches the data-generating process and therefore provides a more accurate reconstruction is shown in panel (**c**). *Middle panels:* **d** networks obtained by considering pairwise correlations above a threshold *t* as edges in the network, as shown in the legend. *Right panels:* e Descriptors – measured from networks obtained through correlation thresholding – as a function of the correlation threshold *t*. The solid horizontal line marks the values obtained for the true network, and the horizontal dashed line the values obtained with the Bayesian inference. The plot on the lower right shows the Jaccard similarity between the true and reconstructed networks, and the vertical line marks, in all figures, the threshold value with the maximum Jaccard similarity.

Yet, despite such limitations being well known in some disciplines, they are often neglected in others, thus leading to several works based on correlation thresholding. In Fig. 2 we illustrate how ill-suited this kind of representation is, with a simple example of an Ising model simulated on a food web^64^. The Ising model is a simple mathematical model of ferromagnetism in statistical mechanics in which each node at a given time is in one of two states {−1, +1}. Interactions across edges cause neighbouring node states to align or anti-align with larger probability. Note that we choose the Ising model for this illustration not due to its realistic nature, but due to its simplicity, and ease of interpretation. After drawing *M* = 10^5^ independent samples from the model at the critical temperature, we compute the pairwise correlations between nodes, and select those above a threshold *t* as the edges of our tentative reconstructed network. As the value of *t* is varied across the entire range, the resulting correlation network never achieves more than a marginal resemblance to the true underlying network. We show further in Fig. 2 that typical network descriptors vary significantly depending on the threshold chosen. While it may be possible to choose a particular value of *t* such that a given descriptor matches the true network, these specific values are not consistent for all descriptors. This inconsistency raises a crucial problem for those studies that select an “optimal threshold” on the basis of this type of analysis, since such optimal threshold changes with the descriptor, thus invalidating the concept of global optimization. Even if the threshold value is chosen to minimize the distance to the true network—an impossible task in practice because the true network is not available—most descriptors still remain severely distorted.

Indeed, the general solution to this problem is to recognise once more the distinction between the data (correlations) and the abstraction (network), and embrace the perspective of inferring, or reconstructing, a hidden network from indirect data. Such ideas are by no means new: Literature on multivariate analysis, as early as the 1970s, frame this problem as *covariance selection*^65^ recognising that conditional independence can be modelled by zeros in the precision matrix (the inverse of the covariance matrix). A classic approach of this kind is the graphical LASSO^66^, which models the set of time series as a multivariate Gaussian and infers a sparse precision matrix that is consistent with the data. We see in Fig. 2 that, even with this misspecified model, we can reconstruct the network much better than naive correlation thresholding.

We can also go further and cast this problem under the exact same inferential framework of Eq. (1), since it represents a very general reconstruction setting. For this problem, the data ***D*** are not noisy network measurements, but instead a set of time series representing the node states evolving over time, i.e., samples from the Ising model^67^. This reconstruction approach has been demonstrated in ref. 68, and we show how it behaves for our example in Fig. 2, where it achieves an accuracy far superior to the threshold approach, for the same data. The reason for this superior performance is straightforward: Eq. (1) allows us to articulate in a formal way what prior knowledge we have about the underlying network and how the data was generated. Eq. (1) also gives us the inference that follows directly from combining these pieces of information. If our assumptions are correct, then we have a provably optimal way to proceed, since no alternative would be able to compete with it. Otherwise, the resulting inference represents the best state of our knowledge, and as soon as a better hypothesis is found, the same framework can be used to compare it with the previous one.

Whatever reconstruction procedure ends up being devised and used in place of the above prescription (even the one where the data is taken “as is”) is formally equivalent to a particular posterior distribution where an implicit model specification does exist but is hidden from view, making it difficult to express or analyze. Nevertheless, its implicit character does not exempt it from justification. Therefore we argue that model specifications need to be made explicit, so that they can be readily judged, rather than protected from scrutiny.

There are no sound scientific arguments for trying to bypass this modelling route, although the difficulty or inability to elaborate on the possible data-generating mechanisms might prove a technical obstacle: e.g., when time series do not come from a known, tractable process such as the Ising model or pulse-coupled oscillators^69^; in such cases any generative model for the dynamics is at best an approximation, and a current research challenge consists in developing a general framework to overcome these limitations. One possibility is to try to infer the dynamical rules together with the network itself, which may be possible to approximate in a nonparametric fashion, using, for example, Bayesian symbolic regression^70^.

(II) Reconstruction based on proximity. The so-called “proximity networks” offer a good example of network analyses where a given observation is mapped directly to an immediate network representation, often bypassing any latent abstraction. In these analyses, time-stamped “edges” are associated with proximity events between two people, i.e., the point in time when they come closer than a pre-defined distance threshold or a face-to-face interaction has been recorded^71–73^.

These important works have allowed a very productive series of studies on temporal networks^74^, by providing a rich set of interpretable network data on which meaningful theories have been developed, as well as the evaluation and comparison of network methodology. Nevertheless, similar to “correlation networks”, it is important to consider that these proximity events may or may not yield the desired information, depending on the context. For instance, physical proximity^73^ may not be so meaningful when reconstructing social relationships. Chance encounters can occur due to shared circumstance, e.g., entering the same elevator, eating in the cafeteria, or sharing public transportation, which are not indicative of meaningful social relationships. Face-to-face interactions, on the other hand, can exclude encounters due to mere proximity^72^, but they still omit the context and nature of the interactions, beyond their time and duration. A more challenging, but arguably more informative approach would be to spell out the different kinds of *latent* pairwise relationships that one would like to consider, and how they affect the proximity events via a generative model (e.g., how differently should close friends interact in space and time when compared to mere acquaintances?). Inferring these types of latent social relationships in this way would be more suitable for most analyses of social dynamics than using the raw spatial data as a proxy.

We emphasize that there is no inherently correct network representation for any particular data, and it all depends on the answers sought. In fact, proximity networks offer an excellent representation in the study of the transmission of infectious disease, for example, which primarily relies on timing and proximity of physical contact, independently on what precipitated the proximity. Well-designed, controlled experiments in which on-person sensors have been carefully selected and calibrated to detect proximity at relevant physical and temporal ranges have been used to reconstruct networks of potential transmission pathways and to study the effect of contagion mitigation strategies^75–83^. Nevertheless, even in such cases where the chosen network representation is convincing, one still needs to consider to what extent it can be extracted from data. The use of heterogeneous or uncalibrated devices, as well as environmental effects (absorption/reflection by objects, walls, floors etc) widens the gap between a detected proximity event and a potential transmission^84^. An appropriate choice of representation never obviates the need for an inferential step that extracts from the data the underlying object of interest, with some amount of uncertainty.

(III) Reconstruction based on specific granularity prescriptions. The choice of the relevant temporal or spatial scale for an analysis, also known as coarse graining, is another ubiquitous and difficult problem to be solved in a variety of applied disciplines, not just network science. A given choice of scale, or aspects of the system that are measured, will invariably hide information at a smaller scale, or aspects that are not measured. An immediate network representation based on a particular choice may therefore provide only an incomplete or distorted picture of the overall system.

A good example is given by spatial networks, such as urban systems or ones at a larger scale. In this case, the source of bias is the modifiable areal unit problem arising when a point-based measure is aggregated into delimited geographic areas^85^. Considering human mobility networks where GPS data is used to infer the flows between distinct parts of a city or a country: point data is aggregated to a chosen granularity in both space and time without a clear clue about which scales have to be used. Unfortunately, this choice can dramatically affect the reconstructed network topology, constraining results to such a specific spatio-temporal granularity and not allowing for an easy generalization of the observed phenomena^86,87^. It is important therefore to recognise the effect of resolution when coarse-graining spatial data in this manner and either take care in selecting an appropriate resolution or explore multiple resolutions in parallel.

There are also examples where the issues of correlations and spacial granularity can even be mixed. A prime example is the reconstruction of the connections in the human brain. One way to approach this is by means of diffusion-based imaging techniques^88^. In this setting, the structural connectivity is inferred from methods like Diffusion Tensor Imaging together with 3D modelling to represent nerve tracts. With this method, the difficulty lies on tracing the neural tracts in a 3D image of objects, and to aggregate the measurements of several individuals. Since the brains of different individuals are not anatomically identical, this requires the use of templates for the atlases used to parcelate the brain. The scale choices involved in this procedure, such as the Regions of Interests (i.e., the nodes), potentially introduce a bias. The output links can either represent the number of tracts or the probability to have those tracts, but the resulting network will depend on the specific method used for modelling and, again, on thresholding choices.

Alternatively, the measurement of the so-called “functional connectivity” is based on fMRI data on local activities on regions of the brain. It also relies on the choice of an atlas to define nodes and the choice of a method to capture correlations between time series, thus potentially introducing a bias due to the choice of one method over another. In this case, the connections represent activity correlations and not physical nerve bundles: network scientists do not consider such connections as part of a tangible network, but a way to map co-activating brain areas under some conditions (e.g., resting state or task performance). We refer to^89^ for a broader and more specialized discussion.

Nevertheless, the result is that topological information inferred using such a heuristic approach depends on the time series length and tends to overestimate certain patterns^90^. These misrepresented patterns have non-trivial consequences for our understanding of the human brain, such as its small-worldness feature^91^, with a strong dependence on the number of parcels^92^. More specifically, Papo et al. have addressed the problem of how network neuroscientists, using standard system-level neuroimaging techniques, can interpret the “small world” construct in the context of functional brain networks, regardless of the fact if the physical human brain is itself a small world network or not, and we refer to their work^91^ for details.

Again, what seems to be lacking is a clear articulation via a generative model (prior to any measurement) of what constitutes the connection between brain regions in the first place, and how that relates to the measured data. Network models applied to functional connectivity are possible, for example, once one frames it as a reconstruction problem, where the features inferred relate to an underlying network responsible for the observed co-activation patterns^68,93^.

(IV) Reconstruction based on state space and temporal correlations. Another reconstruction approach consists of considering an observed time series as the outcome of an (unknown) dynamical system that can be written in general as as

3 $${\dot{x}}_{i}(t)={F}_{i}(x)+{\xi }_{i}(t),$$

where *x*_*i*_(*t*) is the state variable of the *i*–th unit in a network of size *N*, *F*_*i*_ encodes nonlinear functions of the state variables and their coupling, *ξ*_*i*_(*t*) is a noise term with zero average, and *x* = {*x*_1_, *x*_2_, . . . , *x*_*N*_} denotes the system’s state vector. This representation allows one to cast the reconstruction task as the solution of an inverse problem, where the theory of dynamical systems provides a set of powerful tools to characterize the underlying dynamics in terms of state space reconstruction and invariant measures.

Perhaps the most paradigmatic examples of this kind of approach stem from the work of Marc Timme and collaborators^94–98^. Largely, these methods are based on finding the minimal network (i.e., smallest *ℓ*_*p*_-norm of the edge couplings) that is compatible with the undetermined inversion of the nonlinear system (often coupled oscillators, but generalizations are possible^99^) given the observed dynamics. This kind of ansatz provides a revealing connection between the theory of dynamical systems and network reconstruction. One drawback, however, is that it provides only *point estimates* of the inferred networks, i.e., a single network estimate with no uncertainty assessment.

Another example comes from applications to atmospheric dynamics, where data is available on a spatio-temporal grid and a combination of dimension reduction and reconstruction is used to test hypotheses about the underlying mechanisms^100^. Another approach, applied on extreme-rainfall events, introduces a technique that corrects for the bias due to multiple comparisons — similar in spirit to what is done to build Bonferroni networks^101^ — and combine it with network analysis, to unravel global teleconnection patterns likely to be controlled by a physical mechanism known as Rossby waves^102^. A suitable combination of a discovery algorithm with conditional independence tests have been recently used to infer networks validated by both synthetic and real-world systems, the latter based on known physical mechanisms in the climate system and the human heart^103^.

In some cases, relationships can be also deduced without explicitly requiring the knowledge of the governing dynamics equations while assuming that nonlinear deterministic dynamics are at work^104^. This is the case of empirical dynamic modelling — accommodating desirable cases such as nonequilibrium and nonlinear dynamics, and can be used for “equation-free” modeling and forecasting^105^, with emerging applications to network dynamics^106^ — as well as other methods based on state-space reconstruction^107^.

Since the complex dynamics of interconnected units can be often described by a set of coupled ODEs where each equation consists of one term encoding an agent’s self-dynamics and one term encoding the influence of other agents, it is possible to approximate a solution to the inverse problem by using complete orthonormal bases. Following this prescription, one can use the observed time series to estimate the unknown coefficients of the model by formulating a linear inverse problem for each agent. This approach turned out to be successful when applied to a broad range of problems, from studying social synchronization in groups of mice to interdependent electrochemical oscillators^108^.

Although the approaches mentioned above come substantially closer to the general inferential procedure we propose, in general they fall short of a complete implementation. This is because they typically avoid an explicit definition of a generative model, and attempt to reconstruct the underlying network from temporal correlations in the time series. Although these approaches are often said to uncover causal relationships, this is in fact a misnomer, and an instance of the *post hoc ergo propter hoc* fallacy of assuming that one event preceding another can be used as a proof of causation. Although, as we already mentioned, there is little hope of completely disambiguating causation purely from observational data, a proper inferential framework will ascribe to every possible set of causal connections compatible with the data an equal posterior probability, provided they are also equally likely a priori. This will convert the causal ambiguity present in the data into an uncertainty estimate, which is something that most of the approaches mentioned above lack, since they yield only a “point estimate,” i.e., a single network with no associated error assessment.

Furthermore, reconstruction approaches that claim to be “model-free” should be met with a degree of scepticism, since this is arguably impossible. This is because we can use Bayes’ formula of Eq. (1) in the reverse direction, and obtain from any inference procedure *P*(***A***∣***D***) a corresponding generative model *P*(***D***∣***A***) that is compatible with it. Therefore, reconstruction approaches cannot be free of models; at most they can be obscured from view behind the technical implementation of the method. This inability to easily scrutinize the unavoidable modelling assumptions that must come with any conceivable reconstruction method renders these allegedly model-free approaches black boxes that should be handled with care. Ideally, the modelling assumptions should be made explicit to invite scrutiny, model selection and the usual iteration of the scientific process.

(V) Reconstruction based on responses to perturbations. In some cases, one observes the time course of a system’s units, but has also the opportunity to either: (i) act on the system with small perturbations and record its dynamical response, or (ii) access data where known perturbations happen at specific points in space and time.

In such scenarios, which can be reliably summarized into controlled perturbation experiments, one can develop ad hoc methods to exploit the system’s response to reconstruct the unknown topology. For instance, it is possible to quantify the asymptotic response after relaxation, where the system has reached a new dynamical state of equilibrium that can be captured by a response matrix^109^. This approach allows one to capture a node’s impact on its local neighbourhood and recover some apparently ubiquitous scaling laws across disparate complex systems, finding applications in cellular dynamics and human dynamics in online social networks^110^.

For specific types of dynamics (e.g., epidemic spreading or information diffusion) perturbations can be encoded at each time step by specifying the change in state of a unit, in response to the corresponding changes in state of its neighbors at previous time steps. In this case, the equations for the underlying dynamics can be written in terms of a typical susceptible-infected-susceptible (SIS) model, by mapping the reconstruction problem to solving a convex optimization problem such as compressed sensing^111^. While this approach is powerful under its working hypotheses, it is limited to the class of considered dynamical systems. Nevertheless, it provides a promising procedure that can be adapted to other inverse problems. In fact, the same trick of transforming the reconstruction problem into one of sparse signal reconstruction can be applied to other contexts, such as evolutionary games and communication processes, where another convex optimization method, namely the LASSO, can even be used in the presence of noisy time series and partially missing node data^112^.

Another possibility is to exploit responses of invariant measures of adequate driving signals for reconstructing physical interactions among system’s units. Under mild assumptions, it can be shown that the vector of averaged driving signals can be directly related to response differences and the Jacobian matrix of the underlying dynamics. The larger the number of controlled perturbation experiments, the higher the accuracy in reconstruction. However, often it is unrealistic to achieve many of such experiments: for this reason, one can exploit compressed sensing to find a solution of the corresponding optimization problem, achieving remarkable accuracy in reconstructing empirical networks, such as the circadian clock network in *Drosophila*^97^. This technique is powerful but its performance might critically depend on the number of available experiments.

## Suitability of the methods

Central to many network science techniques is the calculation of network descriptors, e.g., degree distribution, clustering, centrality, maximum modularity^1^. The aim of collecting these descriptors is that they might provide a meaningful summary of the properties of the network, such as identifying influential nodes with respect to some measure of importance or characterizing small-scale and meso-scale structures.

However, it is not uncommon that descriptors are employed in a black-box manner, without adequately considering their original context, limitations and interpretability, and are simply presented with little discussion on the implication of the reported values. This kind of careless application of network descriptors means that even when we understand what a particular descriptor is measuring, that descriptor may not provide meaningful information in a particular context. For example, a descriptor may lose its applicability due to the underlying statistics, such as when one summarizes a degree distribution with the mean degree, despite the former being heavy-tailed, and hence rendering the mean unrepresentative. Inapplicability also occurs due to basic problems of interpretation, e.g., when descriptors such as shortest paths no longer carry the same meaning when applied to networks whose edges represent statistical correlations or probabilities.

In the following, we highlight some common problems that arise when network descriptors are employed in an inappropriate manner.

(I) Formulating null models and testing hypotheses. Some descriptors measure a deviation from the mean of a randomized null model, such as modularity^113^—a measure of the tendency of a network to be organized into assortative groups—and the rich club coefficient^114^—a measure of the tendency of highly linked nodes to connect with each other. Such descriptors consequently have a number of assumptions baked in (i.e., those of the null model), which almost certainly will not be universally appropriate and are too often overlooked.

These network descriptors are context-dependent such that their absolute value cannot be interpreted directly, e.g., a network with a maximum modularity of *Q* = 0.9 does not necessarily have a stronger community structure than another with *Q* = 0.6 (in fact, even networks which are completely random can have high modularity values^115^). Therefore, it is often necessary to frame the observed value against the distribution of values under an explicitly stated and suitably chosen null model. The use of a null model in this way requires a decision about what elements of the network should be randomized, but how to make this decision is still largely an open question. Key to answering it is determining which properties of the network are important to a given problem, and therefore need to be fixed, and which properties can be varied. For example, in the pursuit of determining whether or not a particular node feature bears relevance to the network structure we see examples based on comparing a network statistic against null distributions that either fix the network structure and permute the node features^116,117^ or fix the degree distribution and rewire the links^118^ or a combination of the two^119^. It remains unclear which null model we should prefer for a given situation. In many cases, attempting to say what the network is not (i.e., choosing a null model) cannot be fully decoupled from assumptions about what the network may be (i.e., a generative model), therefore attempting to do the first as a means of fully bypassing the second goal is generally ill-fated.

**Fig. 3:**
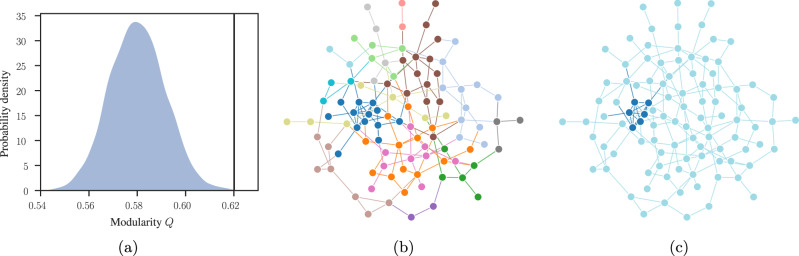
Evaluating community structure with null and generative models. **a** Distribution of maximum modularity value *Q* for a null model of a fully random network with *N* = 100 nodes. The vertical line marks the value encountered for a modified version of the network with an embedded clique of 6 nodes, as described in the text. **b** Consensus partition found by maximizing modularity on the modified network with a planted clique. **c** Partition inferred by fitting the SBM, for the same network as in **b**.

Furthermore, while testing against null models can be good practice when it allows us to easily exclude well-defined scenarios that lack a particular property of interest, one can argue it is fundamentally misguided whenever the answer we are trying to extract from data requires more than a single scalar value. When we try to reject a null model, we can only answer the question of which model was not responsible for the data. When our hypothesis involves detailed multi-dimensional structures, this answer is irrelevant at best. In the worst case, it induces a serious but common misconception that the absence of evidence for the null model implies evidence for the existence of another hypothesis that has not been explicitly tested. A prominent example of this error is community detection using modularity maximization^120^. Although the many serious drawbacks of this nonstatistical approach have been long identified and studied in theoretical and methodological papers^115,121–123^, modularity maximization is still widely employed, especially in domain-specific applications. While the modularity value itself measures a deviation from a null model, when it is maximized it does not possess statistical regularization, and therefore it finds high-scoring partitions even in completely random graphs, which are entirely due to random fluctuations^115,124^. This has led many researchers to suggest computing the statistical significance of the modularity value, when compared to a null model of a completely random network^125^. It is easy to see, however, that framing the problem in this way is in fact inappropriate. We illustrate this in Fig. 3, where we show a completely random graph, with the addition of a few non-random edges forming an embedded clique composed of 6 nodes. Despite being very small, this modification of the network is very unlikely given the null model, and therefore it causes the value of maximum modularity to deviate significantly from what is obtained from the null model. When encountering such a network, we would therefore confidently conclude—correctly—that the null model of a fully random network should be rejected, as we see in Fig. 3a. However, this does not mean that the community structure found—the actual partition of the nodes—is statistically meaningful. We can see this by inspecting the actual partitions found, as shown in Fig. 3b. We see that although the high-scoring values of modularity correspond to partitions where the planted clique is, to some extent, identified, it also finds numerous other communities that bear no relevance to the actual generative process behind the network, i.e., they represent density fluctuations that arise directly from the randomness of the null model^126^.

The reason why the above answer is inappropriate is because we are asking the wrong question: it is largely irrelevant if the maximum value of modularity is statistically significant, what matters is to what extent the partitions found can be attributed to the null model. To answer this question, it is substantially more productive to in fact flip it around, and try to determine which model is more likely to be responsible for the data, rather than which null model should be rejected. This idea forms the backbone of the modern inferential approaches based on the stochastic block model (SBM)^126–129^, which ascribe to each possible network partition ***b*** a posterior probability

4 $$P({{{{{{{\boldsymbol{b}}}}}}}}|{{{{{{{\boldsymbol{A}}}}}}}})=\frac{P({{{{{{{\boldsymbol{A}}}}}}}}|{{{{{{{\boldsymbol{b}}}}}}}})P({{{{{{{\boldsymbol{b}}}}}}}})}{P({{{{{{{\boldsymbol{A}}}}}}}})},$$

where *P*(***A***∣***b***) is the SBM likelihood, a generative model for the network structure that takes into account its modular structure. As we see in Fig. 3c, when applied to the same network, this approach has no trouble in not only perfectly identifying the planted clique, but in also correctly determining that the remaining nodes belong to the same partition, meaning they all have the same probability of connecting to the rest of the network. This method works better because it amounts to asking a more appropriate and fundamental question: what is the most likely division of the network into groups? It is not possible to answer this question in any way other than through Eq. (4); although we can choose its parts in many ways^129^. Incidentally, with this approach we are also able to reject the null model of a fully random network even more explicitly than via modularity. The reason is because a fully random network amounts to a special case of the SBM with a single group, for which we are able to write down an exact posterior probability. Nevertheless, despite its conceptual, theoretical and practical superiority this kind of approach has not yet reached some domains of application.

By asking ourselves why these approaches have not permeated the full breadth of network science domains, we can identify a number of possibilities. One particular issue is that researchers are subject to the incentive to search for the next big breakthrough rather than explore the equivalences between existing methods^130,131^, or revisit prior work or established beliefs (e.g., node attributes are not true communities^132^, network data are uncertain^43^ and thresholding correlation matrices is futile^65^) and potentially reinvent the wheel^133^. Community detection provides a particular case in point, as the SBM was first developed decades ago^134,135^ (although robust and efficient methods for its inference have only been developed in the last decade). Since the introduction of the community detection problem^136^ we have seen a very large amount of methods developed, creating a lack of coherence in theory. An unfortunate outcome when many of these methods can be seen as less principled variants or approximations of the SBM framework^130^. This over-exploration of a particular part of the problem space has almost certainly come at the cost of neglecting other related problems of network clustering^126,137^.

(II) Accounting for reconstruction uncertainty. There are other practical cases where one asks the wrong questions of the data. Since any real-world measure is affected by measurement errors, it should be standard practice to account for them, as well as their propagation, when estimating network descriptors. This would allow us to quantify the uncertainty of our estimation and to compare results across different measurements and experiments, as in any quantitative discipline. Conversely, the lack of such a suitable procedure inevitably leads to estimations that cannot be compared across experiments and, consequently, to conflicting outcomes that cannot be easily resolved.

**Fig. 4:**
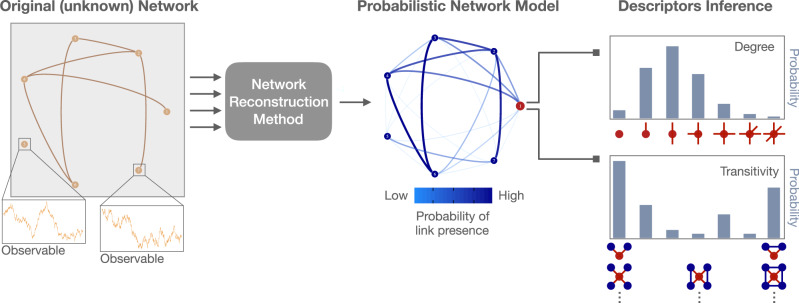
Measuring network descriptors under uncertainty. In many practical problems, the structure of a network cannot be directly observed, while signals (e.g., the time course of physical variables) can be measured from its units. After applying a valid reconstruction method as the ones described in Sec. (II), the results can be summarized in a convenient way via a marginal probability of the existence for each pairwise link, at the expense of some loss of information present in the full joint distribution. Note that the set of those probabilities does not represent a weighted or directed network, and it would be wrong to perform any standard network analysis on the corresponding graph. In fact, in this context, single measures at the level of a single node or the whole network are replaced by probability distributions, encoding how likely it is to measure a specific value. For instance, in a network of size *N*, instead of the degree *k*_*i*_ of a single node *i* one estimate the probability *P*(*k*_*i*_ = *k*) that that node has degree *k*, with *k* = 0,1,...,*N*. Similarly, the transitivity *c*_*i*_ of node *i* is replaced by the probability *P*(*c*_*i*_ = *c*) that that node has transitivity *c*, which might correspond to multiple local configurations rather than a specific one, as illustratively shown in the right-hand side of the figure. Such probability distributions might be obtained within the framework of Bayesian inference used to build the probabilistic network model^106^.

Let us consider again the scenario where the underlying network structure is not known, but it is possible to measure some signals from the nodes (see Fig. 4). Very often correlation or causal analysis is performed to represent the functional relationships between a system’s units and from this representation, network descriptors are successively calculated.

After applying a valid reconstruction method, as the ones described in Sec. (II), it is also possible to summarize in a convenient way the marginal probability *p*_*ij*_ of existence of an edge between nodes *i* and *j*, conditional on the available data and at the expense of some loss of information present in the full joint distribution. This step might be necessary in some situations, e.g., when a plausible model for the dynamics is still not clear.

The outcome is a probabilistic network that, however, cannot be simply analysed as a traditional network since probabilities do not represent traditional weights. Note that a probabilistic network model is the outcome of any valid reconstruction method in the considered scenario: there is no way to upgrade a link measured from a statistical procedure to a real link.

In this case, no network descriptor can be correctly represented by just one number. Let us consider, for example, the degree, the simplest node centrality measure which counts the number of its incoming and out-going links. If each link has a certain probability of existing, the degree itself becomes a stochastic variable which requires a probability distribution to be described. Therefore, reporting that *i* has degree equal to 3, for instance, is meaningless for systems reconstructed from the observation of its units’ signals: instead, it makes sense to assess that the probability that node *i* has degree 3 is equal to 10% or to assess that it is 3 within a confidence or credible interval. While this assessment might appear natural to a statistician, it is still rather overlooked. Methods facilitating this type of estimation are still poorly developed and appearing only recently^106^, focusing on specific problems such as networks of coupled oscillators^138^, redefining nulls models by using random matrix theory^139^ or proposing ad hoc configuration models able to preserve node strength^140^. However, reconstruction based on Bayesian inference provides an elegant and unifying framework that also naturally allows us to accommodate such requests.

## Outlook

The great promise of network science, built on decades of shared efforts across a broad spectrum disciplines to develop a common language, is accompanied with the responsibility to further advance such a language beyond the intrinsically limited perspective of each discipline in isolation. The benefit in the consolidation of network science is not limited to its own community: in fact, requiring enhanced methodological standards catalyzes the cross-pollination between distinct disciplines.

Therefore, for the near future, it is important to not lose sight of the need for rigorous theory and methods, their careful application, and the prudent interpretation of their results. Network science is not about constructing arbitrary networks and calculating tables of network statistics. Instead it should be a comprehensive framework that adds value, allows us to test new hypotheses and ascertain new insights and interpretations: together, methods and applications have shaped and will continue to shape network science.

To realise its full potential, it is essential to preserve and strengthen the link between methods and applications by recognizing the interdisciplinary nature of network science. Collecting scientists from a broad range of backgrounds presents a tremendous opportunity for cross-fertilization of ideas, but that only works if we all understand each other. To strengthen the links between theory and applications it is imperative that we establish a shared epistemological understanding that is reflected in our methodology and common language. We should also be wary of simply transposing terms from one domain onto another, e.g., a network community does not necessarily imply a social or ecological community^117^, network controllability does not necessarily imply that we can perform mind control^141^.

Looking forward, we require more critical thought about the choices we make in applying network methods. Network science is a relatively young discipline, but one that is built upon the strong foundations of well-established methodological disciplines such as mathematics, physics and computer science as well as all the application domains where it is employed. As it moves towards a new stage in its maturity, the field needs to consolidate best practices, in the same way it happened and it continued happens in other mature fields. For instance, it is desirable to avoid purely heuristic (re)construction of networks and the application of network statistics without caution, following good practices as the ones discussed in this work.

As attractive as it sounds that we can represent so many complex systems as networks to make use of a common set of tools, ultimately we should be moving away from creating one-size-fits-all solutions. Instead, we should be working more closely together to develop rigorous methods that incorporate relevant domain knowledge and allow us to probe more deeply and directly address the questions that can only be answered by viewing the system as a whole.

Here we have demonstrated how generative models provide promising means to progress towards these common goals. Generative models are extensible and can be easily adapted to explicitly encode specific hypotheses and assumptions about complex systems and how we observe them. Statistical inference allows us to fit these models and compare levels of support for competing hypotheses. Developing end-to-end models in this manner acknowledges that the process of network analysis, from data to conclusion, is itself a complex system. No stage in the analytical process is independent and therefore should not be handled in isolation. Our design decisions across the whole of the analytical process should be connected such that specific choices can inform one another^54,68,93,142^.

Based on our analysis, we can identify a succinct set of best practices for the next advances of network science and its applications to domain-specific challenges. First, we must understand the provenance of network data and make explicit, via a generative model, the underlying abstraction that one wishes to extract from it. Ideally, our chosen abstraction should not depend on an arbitrary choice of spatial and/or temporal granularity, or we should at least demonstrate that the resulting analysis is not sensitive to this choice. Second, we must incorporate, both theoretically and in practice, the presence and the effects of errors and incompleteness in network measurements. Third, when developing a new analytical approach, we should validate it on synthetic data to guarantee that expected outcomes are found. If the validity of a method cannot be demonstrated on controlled synthetic experiments, results obtained when it is applied to empirical data have little value.

Much of what we are discussing may appear standard and established guidelines to the eyes of researchers in particular empirical scientific areas. What we stress here is that there are subtle issues that can emerge when rigorous methodologies are not employed more generally. The network science community is characterized by the heterogeneous background of its members — one of our community’s strengths. However, such heterogeneity also means that many of the above points, as a whole, are not universally recognized, and therefore require emphasis. For instance, methodological flaws that are easily identified within the boundaries of a specific discipline can inevitably lead to controversial results even when applied to the same data, within the wider boundaries of network science. Therefore, it is necessary to move beyond the particular languages and practices of individual disciplines and positively cross-pollinate to obtain a shared standard of best practices across our multidisciplinary field.

Only when used responsibly, and with the appropriate level of methodological rigour, can the tools of network science give us unique insight into the structure, function and dynamics of complex systems.
